# Supplementary material for: Drone honey bees are disproportionately sensitive to abiotic stressors despite expressing high levels of stress response proteins
Source: Commun Biol. 2022 Feb 17;5:141. doi: 10.1038/s42003-022-03092-7 (PMC8854713; doi:10.1038/s42003-022-03092-7)
Supplement: Supplementary file 2 — Description of Additional Supplementary Files [file 42003_2022_3092_MOESM2_ESM.pdf]

Description of Additional Supplementary Files

File name: Supplementary Data 1-6
Description:

Supplementary Data 1: Underlying topical pesticide exposure and cold exposure survival data.

Supplementary Data 2: Underlying drone morphometric data.

Supplementary Data 3: Survival data associated with colony exposures.

Supplementary Data 4: Proteomics data of drones and workers exposed to acetone, imidacloprid, and cocktail treatments.

Supplementary Data 5: Summary statistics of Supplementary Data 4.

Supplementary Data 6: Proteomics data of untreated workers and drones.
