# Supplementary material for: Drone honey bees are disproportionately sensitive to abiotic stressors despite expressing high levels of stress response proteins
Source: Commun Biol. 2022 Feb 17;5:141. doi: 10.1038/s42003-022-03092-7 (PMC8854713; doi:10.1038/s42003-022-03092-7)
Supplement: Supplementary file 4 — Reporting Summary [file 42003_2022_3092_MOESM4_ESM.pdf]

nature research

Corresponding author(s): Alison McAfee

Last updated by author(s): Dec 21, 2021

Reporting Summary

Nature Research wishes to improve the reproducibility of the work that we publish. This form provides structure for consistency and transparency in reporting. For further information on Nature Research policies, see our Editorial Policies and the Editorial Policy Checklist.

Statistics

For all statistical analyses, confirm that the following items are present in the figure legend, table legend, main text, or Methods section.

n/a	Confirmed	
☐	☒	The exact sample size (*n*) for each experimental group/condition, given as a discrete number and unit of measurement
☐	☒	A statement on whether measurements were taken from distinct samples or whether the same sample was measured repeatedly
☐	☒	The statistical test(s) used AND whether they are one- or two-sided<br>*Only common tests should be described solely by name; describe more complex techniques in the Methods section.*
☐	☒	A description of all covariates tested
☐	☒	A description of any assumptions or corrections, such as tests of normality and adjustment for multiple comparisons
☐	☒	A full description of the statistical parameters including central tendency (e.g. means) or other basic estimates (e.g. regression coefficient) AND variation (e.g. standard deviation) or associated estimates of uncertainty (e.g. confidence intervals)
☐	☒	For null hypothesis testing, the test statistic (e.g. *F*, *t*, *r*) with confidence intervals, effect sizes, degrees of freedom and *P* value noted<br>*Give P values as exact values whenever suitable.*
☒	☐	For Bayesian analysis, information on the choice of priors and Markov chain Monte Carlo settings
☒	☐	For hierarchical and complex designs, identification of the appropriate level for tests and full reporting of outcomes
☒	☐	Estimates of effect sizes (e.g. Cohen's *d*, Pearson's *r*), indicating how they were calculated

Our web collection on statistics for biologists contains articles on many of the points above.

Software and code

Policy information about availability of computer code

Data collection	MaxQuant (v 1.6.1.0)
Data analysis	Perseus (v 1.6.1.1) and R (v 3.6.0 and 4.0.2) were used to analyze the data. All software and R codes are available upon request. Previous versions of R are available at https://cran.r-project.org/bin/windows/base/old/

For manuscripts utilizing custom algorithms or software that are central to the research but not yet described in published literature, software must be made available to editors and reviewers. We strongly encourage code deposition in a community repository (e.g. GitHub). See the Nature Research guidelines for submitting code & software for further information.

Data

Policy information about availability of data

All manuscripts must include a data availability statement. This statement should provide the following information, where applicable:

- Accession codes, unique identifiers, or web links for publicly available datasets
- A list of figures that have associated raw data
- A description of any restrictions on data availability

Topical pesticide exposure and cold exposure survival data are available in Supplementary Table S1. Drone morphometric data are available in Supplementary Table S2. Survival data associated with colony exposures are available in Supplementary Table S3. Proteomics data of drones and workers exposed to acetone, imidacloprid, and cocktail treatments are available in Supplementary Table S4, and summary statistics of these data are available in Supplementary Table S5. Proteomics data of untreated workers and drones are available in Supplementary Table S6. All raw mass spectrometry data are available on MassIVE (www.massive.ucd.edu, accession: MSV000087818). Any codes associated with this manuscript are available from the authors upon request.

Field-specific reporting

Please select the one below that is the best fit for your research. If you are not sure, read the appropriate sections before making your selection.

☒ Life sciences ☐ Behavioural & social sciences ☐ Ecological, evolutionary & environmental sciences

For a reference copy of the document with all sections, see nature.com/documents/nr-reporting-summary-flat.pdf

Life sciences study design

All studies must disclose on these points even when the disclosure is negative.

Sample size	No sample size calculation was performed. We chose sample sizes which were consistent with our previous proteomic experiments investigating effect of stressors in honey bees.
Data exclusions	Proteins only identified by site, potential contaminants, and reverse hits were removed, followed by proteins identified in fewer than three replicates of each group. This reduces the frequency of spurious identifications.
Replication	We replicated the drone vs. worker control comparison, which was central to our conclusions, and the results were consistent with what we previously observed. These results are described in the text.
Randomization	Drones and workers were randomly allocated to their experimental groups. Sample orders were randomized ahead of LC-MSMS injection.
Blinding	Investigators were not blinded, as the endpoints measured were either binary (dead/alive) or not sensitive to user bias (proteomic sample preparation)

Reporting for specific materials, systems and methods

We require information from authors about some types of materials, experimental systems and methods used in many studies. Here, indicate whether each material, system or method listed is relevant to your study. If you are not sure if a list item applies to your research, read the appropriate section before selecting a response.

Materials & experimental systems

n/a	Involved in the study	
☒	☐	Antibodies
☒	☐	Eukaryotic cell lines
☒	☐	Palaeontology and archaeology
☐	☒	Animals and other organisms
☒	☐	Human research participants
☒	☐	Clinical data
☒	☐	Dual use research of concern

Methods

n/a	Involved in the study	
☒	☐	ChIP-seq
☒	☐	Flow cytometry
☒	☐	MRI-based neuroimaging

Animals and other organisms

Policy information about studies involving animals; ARRIVE guidelines recommended for reporting animal research

Laboratory animals	None
Wild animals	None
Field-collected samples	Honey bees were sampled from colonies maintained using standard best management practices at the University of British Columbia and North Carolina State University.
Ethics oversight	As non-cephalopod invertebrates, honey bees are not subject to Animal Ethics oversight at the University of British Columbia and North Carolina State University

Note that full information on the approval of the study protocol must also be provided in the manuscript.
